# Usefulness of 5-ALA (Gliolan®)-derived PPX fluorescence for demonstrating the extent of infiltration in atypical meningiomas

**DOI:** 10.1007/s00701-014-2148-z

**Published:** 2014-06-28

**Authors:** Eike Wilbers, Gunnar Hargus, Johannes Wölfer, Walter Stummer

**Affiliations:** 1 Klinik und Poliklinik für Neurochirurgie, Universitätsklinikum Münster, Albert-Schweitzer-Campus 1, 48149 Münster, Germany; 2 Institut für Neuropathologie, Universitätsklinikum Münster, Albert-Schweitzer-Campus 1, 48149 Münster, Germany

Dear Editor,

Five-aminolevulinic acid-induced tumor fluorescence is receiving increasing attention as a surgical tool after the first reports describing its use in high grade gliomas 1998 [10–13]. Approval in the European Union was based on a consecutive phase III trial in 322 patients which showed a higher rate of complete resections and 6-month progression-free survival in patients operated on using 5-ALA [11]. Meningiomas, benign as well as atypical subtypes, have also been observed to accumulate fluorescence in response to 5-ALA [1–7]. Specifically, Coluccia et al. showed 5-ALA to induce porphyrin fluorescence in 31 of 33 patients with meningiomas (32 of them WHO I-II, one WHO III) [2]. Even for grade I meningiomas of the sphenoid bone, accumulation of protoporphyrin IX (PpIX) fluorescence has been described. Quantitative detection of protoporphyrin IX concentrations using spectrography allowed more accurate detection of the extent of tissue infiltration by meningioma cells in the case of a 52-year-old patient [1]. Kajimoto et al. reported on the usefulness of 5-ALA-induced fluorescence for the detection of residual cells of atypical meningiomas in the dura [6], and the use of 5-ALA for the resection of grade II meningiomas with osseus infiltration has been pointed out as well [8]. Hefti et al. compared the accumulation of PpIX in two human meningioma cell lines (HBL-52 and BEN-MEN-I) and observed PpIX concentrations increased to up to five times normal level in BEN-MEN-1 cells (while the ferrochelatase activity was 2.7 times higher in HBL-52 cells), with a much higher sensitivity to photodynamic therapy (PDT) [4]. PDT relies on the interaction of laser light and porphyrin IX, which, apart from its fluorescence, is also a potent photosensitizer.

Atypical meningiomas have a high recurrence rate of 41 % in five years [5], possibly because the true extent of infiltration of adjacent tissue is not distinguishable, and residual tumor is missed surgically.

**Fig. 1:**
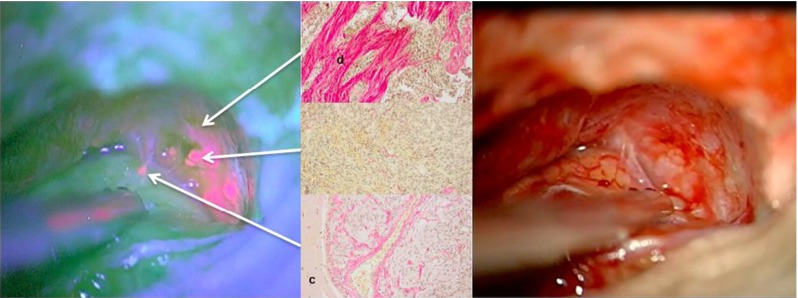
**Left**: Red fluorescent atypical meningioma after 5-ALA with fluorescence in tumor, adjacent dura and arachnoid overlying cortex. **Right**: Corresponding white light image. **Center**: Elastica-van-Gieson staining of biopsies. *Top*: Adjacent, fluorescing dura (“d”); *middle*: gross tumor and *bottom*: invaded brain (“c”: cortex)

With this letter we would like to share our experience regarding a 21-year-old young female patient with atypical meningioma in whom brain dura and brain infiltration could be well visualized using 5-ALA. This patient presented with recurrent right-sided, parasagittal, postcentral atypical meningioma (WHO grade II) initially resected five years earlier and adjuvantly treated by radiosurgery (18 Gy). Resection was performed using 5-ALA (Gliolan®, 20 mg/kg). Fluorescence was noted in the gross tumor. However, several small areas of distinct fluorescence were also observed within the adjacent dura and, importantly, also in the arachnoid of the adjacent cortex. Histological examination revealed meningothelial tumor cells in both the cortex and the brain (Fig. 1).

Thus, 5-ALA (Gliolan®)-derived tumor fluorescence appeared useful for detecting these areas of residual infiltration in the dura but more importantly, also in the adjacent brain, which could be the origin of further recurrences; this has not previously been reported. This observation points out that the adjacent arachnoid may in fact be the source of some tumor recurrences despite macroscopically complete resections of gross tumor and the adjacent dura. Infiltrative growth of meningiomas has been identified as an important factor driving prognosis in patients even with grade I meningiomas [9], and gross total resection of malignant meningiomas has been associated with improved survival. Thus, 5-ALA-derived tumor fluorescence may help in improving the prognosis in patients with invasive but otherwise benign meningiomas, as well as in patients with atypical or anaplastic meningiomas. The detection of PpIX in the infiltrating tumor may also provide a rationale for adjuvant PDT in these lesions.
